# Effect of metformin treatment and its time of administration on joint capsular fibrosis induced by mouse knee immobilization

**DOI:** 10.1038/s41598-021-97445-7

**Published:** 2021-09-09

**Authors:** Kotaro Tokuda, Yoshiaki Yamanaka, Yosuke Mano, Manabu Tsukamoto, Takafumi Tajima, Hitoshi Suzuki, Makoto Kawasaki, Soshi Uchida, Eiichiro Nakamura, Ke-Yong Wang, Akinori Sakai

**Affiliations:** 1 Department of Orthopaedic Surgery, University of Occupational and Environmental Health, 1-1 Iseigaoka, Yahatanishi, Kitakyushu, Fukuoka 807-8555 Japan; 2 Department of Orthopaedic Surgery and Sports Medicine, Wakamatsu Hospital of the University of Occupational and Environmental Health, Kitakyushu, Japan; 3 Shared-Use Research Center, University of Occupational and Environmental Health, Kitakyushu, Japan

**Keywords:** Drug therapy, Experimental models of disease

## Abstract

Joint contracture leads to major patient discomfort. Metformin, one of the most extensively used oral drugs against type 2 diabetes has recently been found to suppress tissue fibrosis as well. However, its role in suppressing tissue fibrosis in joint contractures remains unknown. In this study, we examined the role of metformin treatment in suppressing joint capsular fibrosis and the most effective time of its administration. Joint capsular fibrosis was induced by immobilizing the knee joints of mice using splints and tapes. Metformin was administered intraperitoneally every alternate day after immobilization. Histological and immunohistochemical changes and expression of fibrosis-related genes were evaluated. Metformin treatment significantly suppressed fibrosis in joint capsules based on histological and immunohistochemical evaluation. Joint capsular tissue from metformin-treated mice also showed decreased expression of fibrosis-related genes. Early, but not late, metformin administration showed the same effect on fibrosis suppression in joint capsule as the whole treatment period. The expression of fibrosis-related genes was most suppressed in mice administered with metformin early. These studies demonstrated that metformin treatment can suppress joint capsular fibrosis and the most effective time to administer it is early after joint immobilization; a delay of more than 2 weeks of administration is less effective.

## Introduction

Although joint immobilization is important for treating musculoskeletal diseases^1,2^, it is the most common cause of joint contracture^3^. Joint contracture is characterized by a restricted range of motion (ROM) in the joints^4^ and limits the patient's activities of daily living by decreasing movement ability and impairing joint function^5,6^. Even a slight loss of about 5° of knee extension leads to difficulties in walking, while a loss of flexion leads to problems with climbing stairs and sitting^6^. Joint contracture, caused during common treatments for musculoskeletal diseases, is a very important issue for orthopedic surgeons as it leads to major patient discomfort.

Physical therapy and surgical release are the widely accepted interventions for the prevention and treatment of joint contracture. However, these treatments are ineffective in some patients^7–9^. Therefore, it is important to establish better prevention and treatment methods for joint contracture.

It has been reported that immobilization-induced joint contractures develop due to both arthrogenic (bone, capsule, synovium, cartilage, and ligament) and myogenic components (tendon and fascia)^10^. The arthrogenic components, particularly the joint capsule, play an important role in joint contractures^11,12^; therefore, fibrosis of the joint capsule is considered as one of its main causes^13^.

Metformin is one of the most extensively used oral anti-diabetic drugs for patients with type 2 diabetes. Recently, some studies have revealed an additional biological effect of metformin as a suppressive agent for tissue fibrosis in organs such as the heart, lungs, kidneys, and skin^14–21^. Most studies on the suppressive effect of this drug on tissue fibrosis have suggested that metformin, which has an AMP-activated protein kinase (AMPK)-activating effect, may suppress tissue fibrosis by inhibiting transforming growth factor beta (TGF-β) signaling^14–16,18,20,21^. As joint contracture is caused by tissue fibrosis in the joint, the role of metformin in suppressing the immobilization-induced joint contracture must be investigated. There are currently no previous reports investigating the effects of metformin on joint contracture. Therefore, in this study, we sought to determine, first, whether metformin suppresses fibrosis of the joint capsule, which is the main etiology of joint contractures, and second, the most effective time to administer metformin to suppress fibrosis of the joint capsule.

## Results

### Establishment of the mouse model of knee contracture to test for the effect of metformin treatment

**Figure 1:**
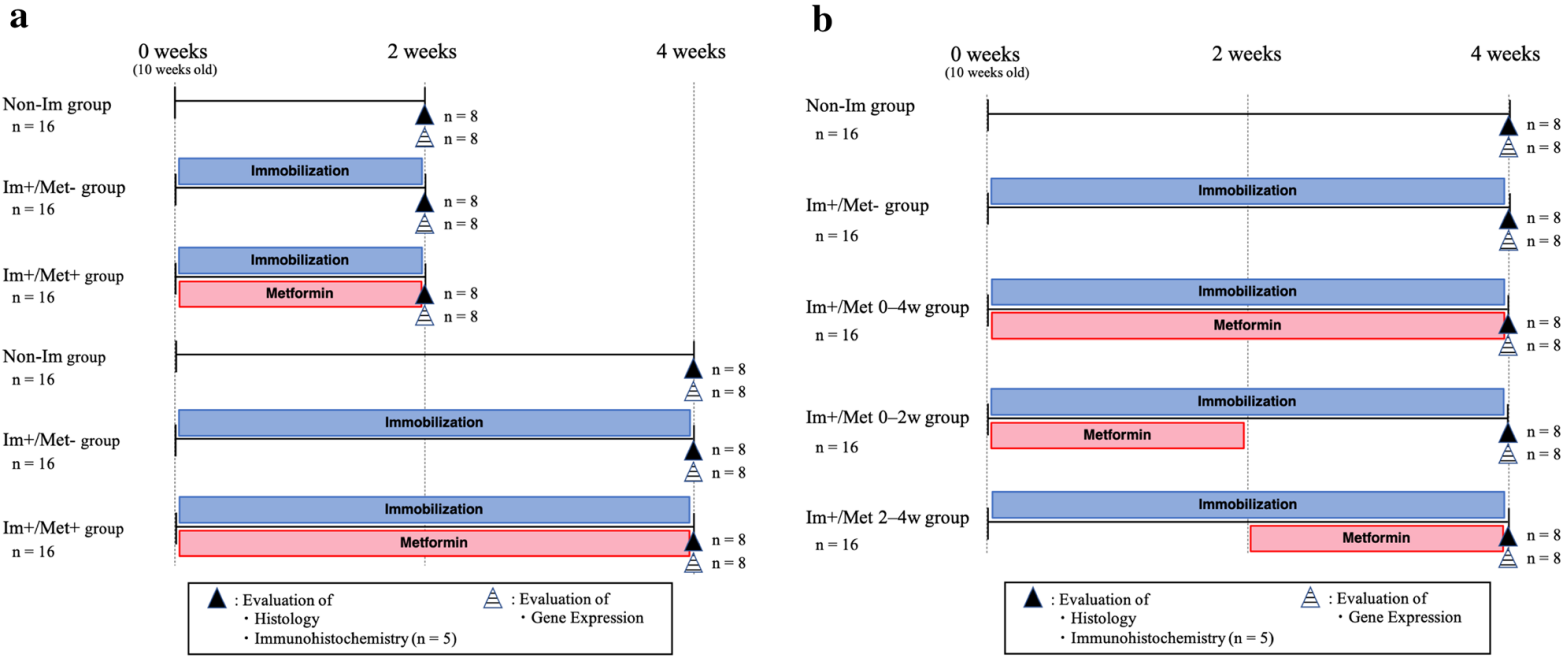
Study flow chart. The flow chart shows the experimental design and number of mice used per experiment/outcome in this study for determining (**a**) the effect of metformin for joint contracture, and (**b**) the effective time of metformin administration for joint capsular fibrosis. Non-striped upward triangles indicate the histological and immunohistochemical analyses. Striped upward triangles indicate gene expression analysis. Non-Im, mice with non-immobilized knees; Im+/Met−, mice with immobilized knees but no metformin; Im+/Met+, mice with immobilized knees who were administered metformin; Im+/Met 0–4 weeks, mice with immobilized knees and metformin administered from 0 to 4 weeks; Im+/Met 0–2 weeks, mice with immobilized knees and metformin administered from 0 to 2 weeks; Im+/Met 2–4 weeks, mice with immobilized knees and metformin administered from 2 to 4 weeks.

The animals were divided into three groups of 32 mice each: (Non-Im), mice with knees not immobilized and metformin not administered; (Im+/Met−), mice with knees immobilized but metformin not administered; (Im+/Met+), mice with knees immobilized and metformin administered (Fig. 1a). During the experimental period, the movement of the right knee joint was restricted for 2 or 4 weeks in selected mice without significant problems or complications. The mice with immobilized knees could move freely in the cage and eat and drink by themselves. Metformin-treated mice also did not have any significant hypoglycemic complications.

#### Histological analysis

**Figure 2:**
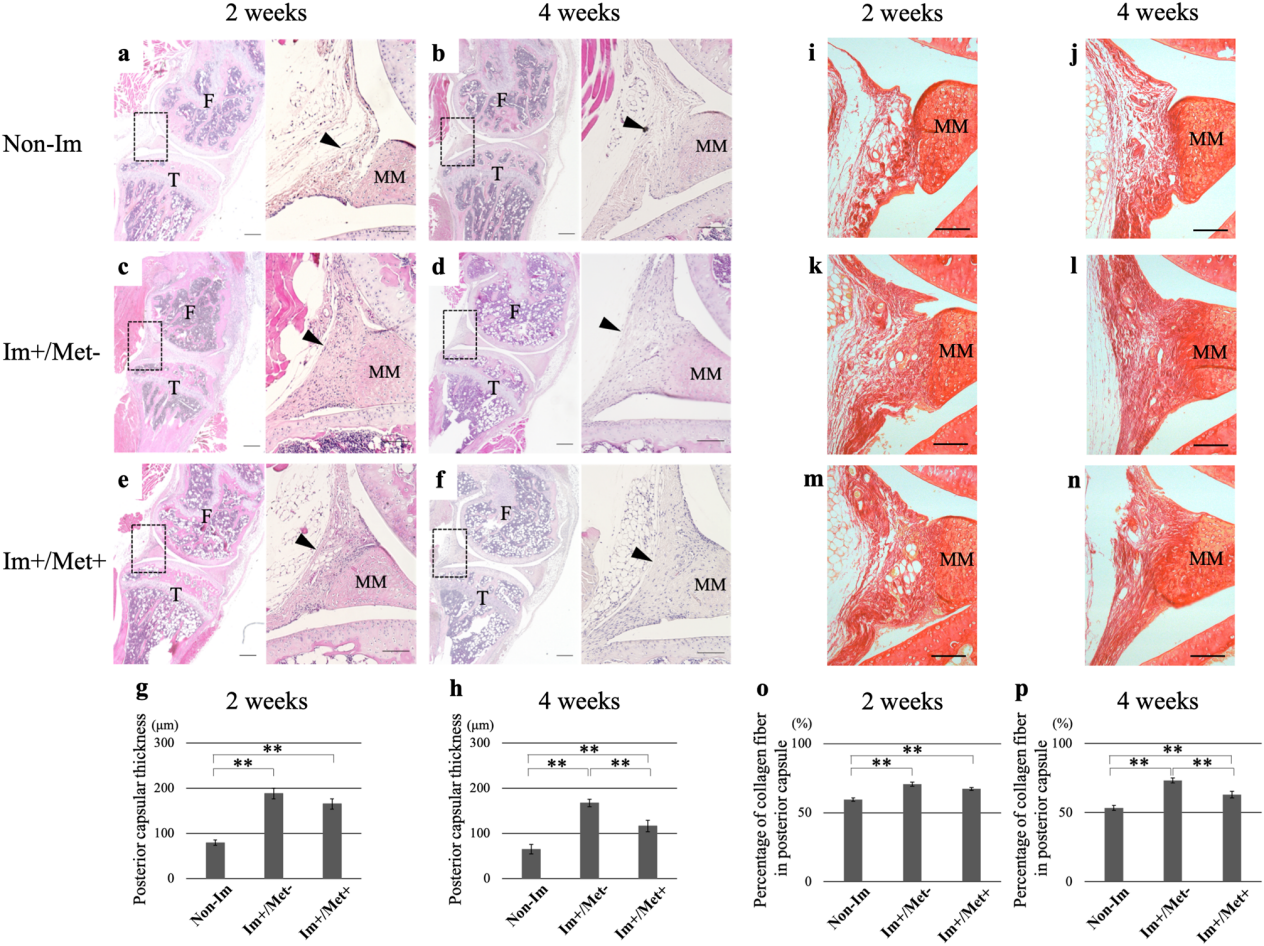
Histological analysis of the effect of metformin on joint capsular fibrosis. Hematoxylin and eosin-stained sections of the knee joints for each group at 2 and 4 weeks (**a**–**f**). The arrowhead indicates the posterior capsule. Each right-side image in (**a**–**f)** shows the high magnification image of the dotted box of its corresponding left side image (Scale bar: 300 μm (right), 100 μm (left)). Posterior capsular thickness at (**g**) 2 weeks and (**h**) 4 weeks. Picrosirius red-stained sections of the posterior joint capsules for each group at 2 and 4 weeks (**i**–**n)** (Scale bar: 100 μm). Percentage of collagen fiber in the posterior capsule at (**o**) 2 weeks and (**p**) 4 weeks measured as the ratios of the total areas stained red by picrosirius red staining to the areas of the posterior capsule. Data are expressed as the mean ± standard errors. **p < 0.01; n = 8. *F* femur, *T* tibia, *MM* medial meniscus, *Non-Im* mice with non-immobilized knees, *Im+/Met−* mice with immobilized knees but no metformin, *Im+/Met+* mice with immobilized knees who were administered metformin.

In hematoxylin and eosin (HE) sections, the posterior joint capsule in the (Im+/Met−) and (Im+/Met+) groups appeared thicker than that in the Non-Im group at 2 and 4 weeks after immobilization (Fig. 2a–f). Quantitative evaluation showed that posterior capsular thickness at both 2 weeks and 4 weeks was significantly greater in the (Im+/Met−) and (Im+/Met+) groups than in the Non-Im group (p < 0.01 in all cases) (Fig. 2g,h). Moreover, posterior capsular thickness at 4 weeks was significantly greater in the (Im+/Met−) group than in the (Im+/Met+) group (p < 0.01).

In the picrosirius red sections, the posterior joint capsule in the (Im+/Met−) and (Im+/Met+) groups appeared to have more densely accumulated collagen fibers than that in the Non-Im group at 2 and 4 weeks (Fig. 2i–n). Quantitative evaluation showed that the percentage of collagen fibers in the posterior joint capsule at both 2 weeks and 4 weeks after immobilization was significantly higher in the (Im+/Met−) and (Im+/Met+) groups than in the Non-Im group (p < 0.01 in all cases) (Fig. 2o,p). Additionally, the percentage of collagen fibers in the posterior joint capsule at 4 weeks after immobilization was significantly higher in the (Im+/Met−) group than in the (Im+/Met+) group (p < 0.01).

#### Gene expression in the joint capsule tissue

**Figure 3:**
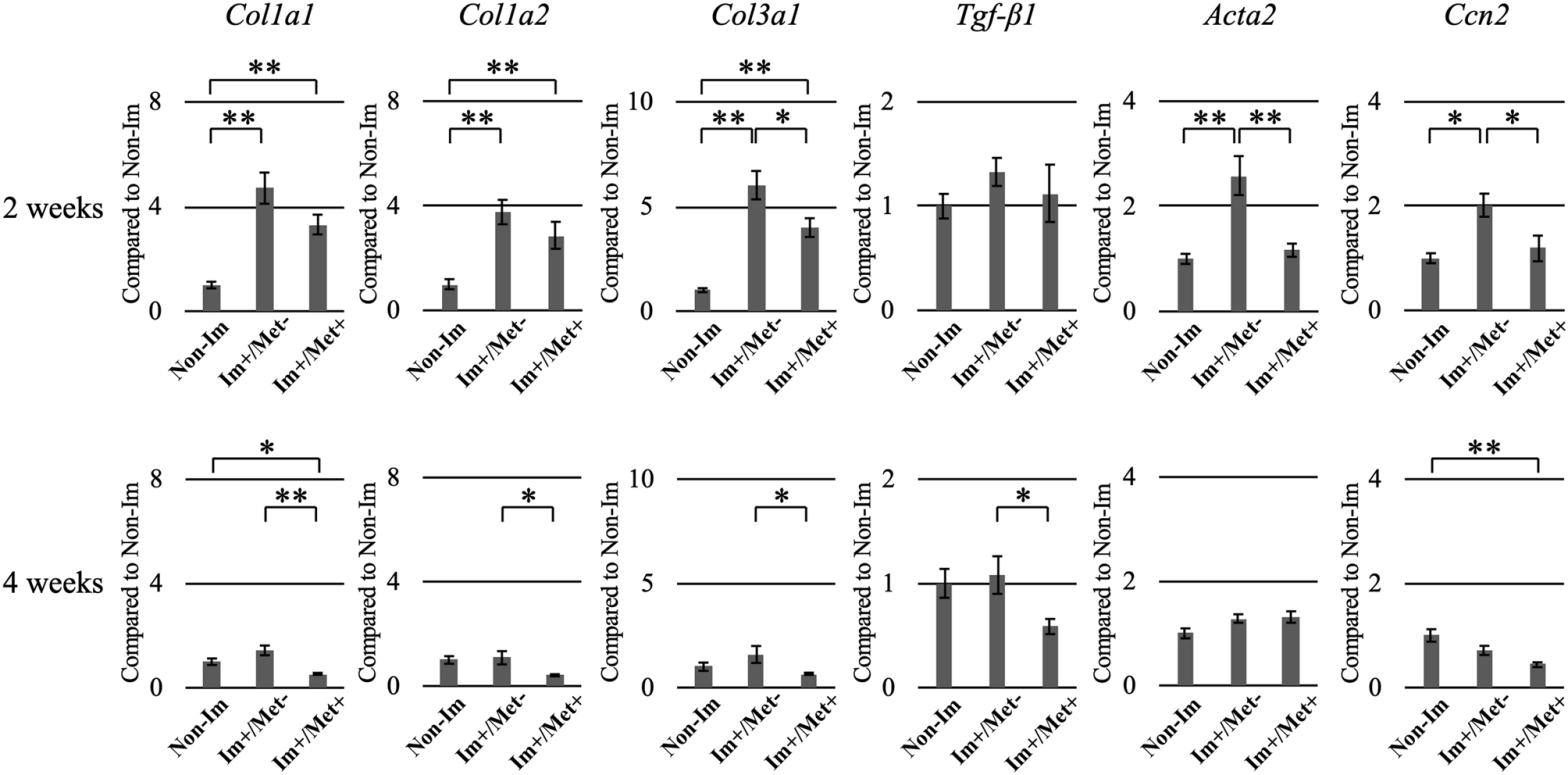
Expression analysis of the fibrosis-related genes in the knee joint capsule for the effect of metformin on joint capsular fibrosis. The mRNA expression of *Col1a1*, *Col1a2*, *Col3a1*, *Tgf-β1*, *Acta2*, and *Ccn2* was normalized to that of *Gapdh*. Data are expressed as the mean ± standard errors. *p < 0.05, **p < 0.01; n = 8. *Col1a1*, collagen type IA1; *Col1a2*, collagen type IA2; *Col3a1*, collagen type IIIA1; *Tgf-β1*, transforming growth factor beta 1; *Acta2*, actin alpha 2; *Ccn2*, cellular communication network factor 2; *Gapdh*, glyceraldehyde-3-phosphate dehydrogenase; Non-Im, mice with non-immobilized knees; Im+/Met−, mice with immobilized knees but no metformin; Im+/Met+, mice with immobilized knees who were administered metformin.

The expression of collagen type IA1 (*Col1a1*), collagen type IA2 (*Col1a2*), collagen type IIIA1 (*Col3a1*), actin alpha 2 (*Acta2*), and cellular communication network factor 2 *(Ccn2*) was significantly higher 2 weeks after immobilization in the (Im+/Met−) group than in the Non-Im group (Fig. 3), but showed no significant differences at 4 weeks. The expression of *Col1a1*, *Col1a2*, and *Col3a1* was significantly higher 2 weeks after immobilization in the (Im+/Met+) group than in the Non-Im group. In contrast, the expression of *Col1a1* and *Ccn2* was significantly lower 4 weeks after immobilization in the (Im+/Met+) group than in the Non-Im group. Additionally, the expression of *Col3a1*, *Acta2,* and *Ccn2* at 2 weeks, and of *Col1a1*, *Col1a2*, *Col3a1* and *Tgf-β1* at 4 weeks was significantly lower in the (Im+/Met+) group than in the (Im+/Met−) group.

#### Immunohistochemical analysis

**Figure 4:**
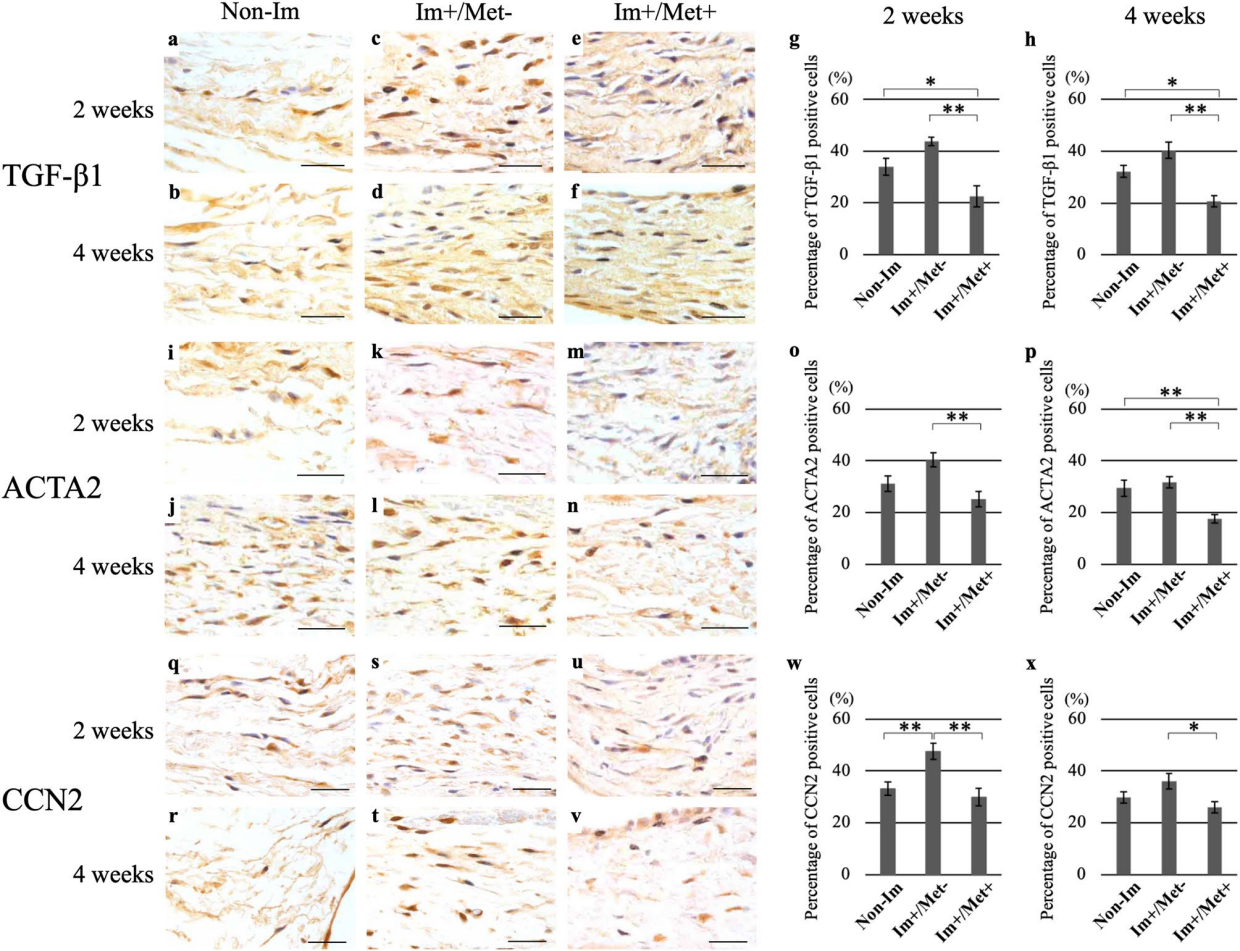
Immunohistochemical analysis for the effect of metformin on joint capsular fibrosis. Immunohistochemical staining for TGF-β1, ACTA2, and CCN2 in the posterior capsule. Microscopic images of specimens immunostained for TGF-β1 (**a**–**f**), ACTA2 (**i**–**n**), and CCN2 (**q**–**v**) with DAB, and counterstained with hematoxylin (Scale bar: 50 μm). Percentage of TGF-β1-positive cells (**g**, **h**), ACTA2-positive cells (**o**, **p**), and CCN2-positive cells (**w**, **x**) to the total number of cells in the posterior joint capsule at 2 and 4 weeks, respectively. Data are expressed as the mean ± standard errors. *p < 0.05, **p < 0.01; n = 5. *TGF-β1* transforming growth factor beta 1, *ACTA2* actin alpha 2, *CCN2* cellular communication network factor 2, *Non-Im* mice with non-immobilized knees, *Im+/Met−* mice with immobilized knees but no metformin, *Im+/Met+* mice with immobilized knees who were administered metformin, *DAB* 3,3′-diaminobenzidine.

As fibroblast-like cells in the posterior capsule were not stained with 3,3′-diaminobenzidine (DAB) by staining with negative control, their staining response was found to be specific. The percentage of CCN2-positive cells in the posterior joint capsule at 2 weeks was significantly higher in the (Im+/Met−) group than in the Non-Im group (p < 0.01) (Fig. 4w). At both 2 and 4 weeks, the percentage of TGF-β1-, ACTA2-, and CCN2-positive cells in the posterior joint capsule in the (Im+/Met+) group was lower than that in the (Im+/Met−) group (Fig. 4g,h,o,p,w,x). The percentage of TGF-β1-positive cells in the posterior joint capsule at 2 and 4 weeks was significantly lower in the (Im+/Met+) group than in the Non-Im group (p < 0.01 and p < 0.01, respectively) (Fig. 4g,h). The percentage of ACTA2-positive cells in the posterior joint capsule at 4 weeks was significantly lower in the (Im+/Met+) group than in the Non-Im group (p < 0.01) (Fig. 4p).

### Effective time of metformin administration for suppressing joint capsular fibrosis

The experimental period was 4 weeks, and the differences among the following five groups were analyzed: a group in which the knees were not immobilized and metformin was not administered (Non-Im), a group in which the knees were immobilized and metformin was not administered (Im+/Met−), a group in which the knees were immobilized and metformin was administered for the whole period (Im+/Met 0–4 weeks), a group in which the knees were immobilized and metformin was administered only for the initial 2 of the 4 weeks (Im+/Met 0–2 weeks), and a group in which the knees were immobilized and metformin was administered only for the final 2 of the 4 weeks (Im+/Met 2–4 weeks) (Fig. 1b). During the experiment, there were no significant complications due to immobilization of the knee joints and administration of metformin.

#### Histological analysis

**Figure 5:**
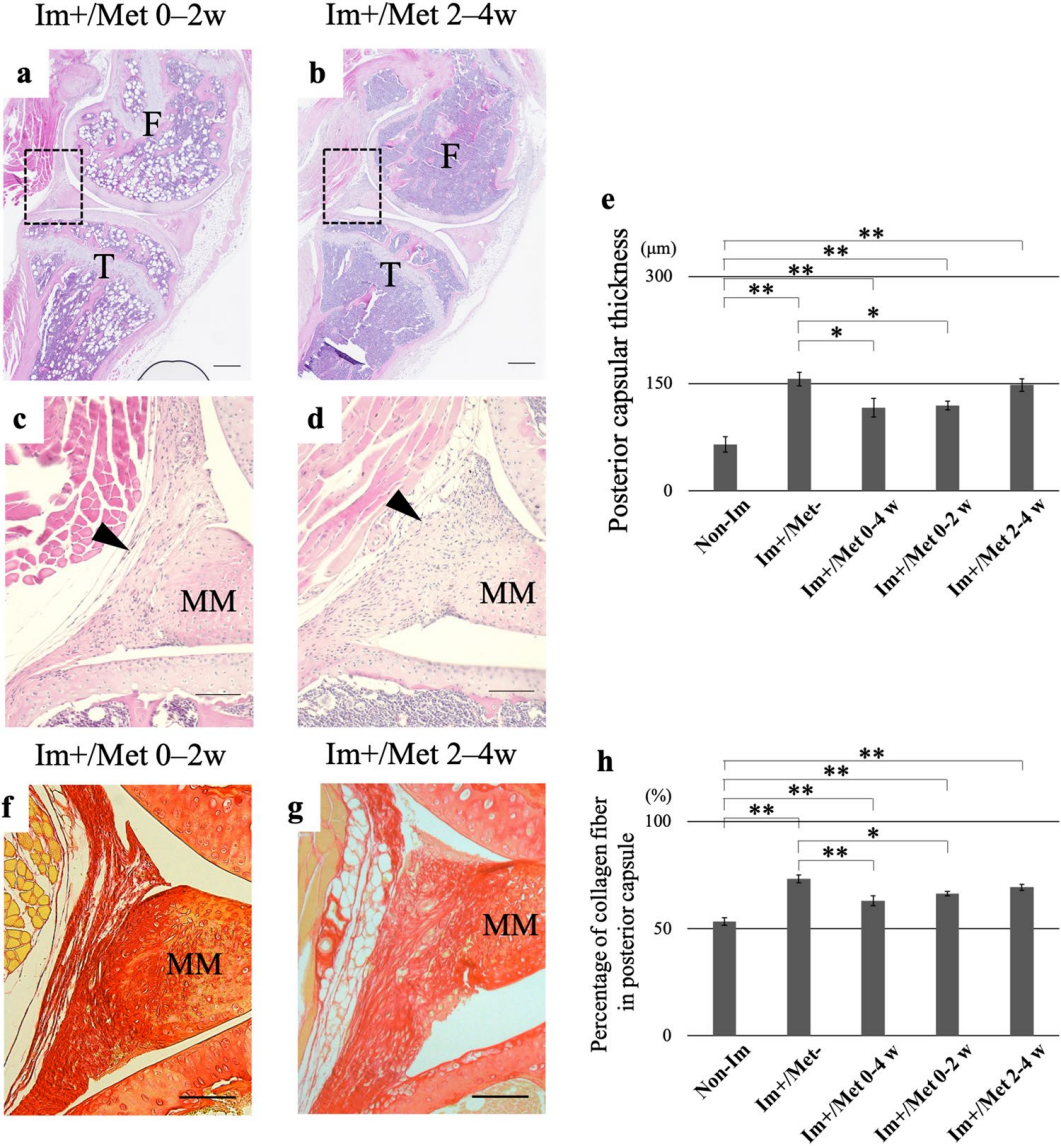
Histological analysis for the effective time of metformin administration for joint capsular fibrosis. Hematoxylin and eosin-stained sections of the knee joints in the (Im+/Met 0–2 weeks) group (**a**) and (Im+/Met 2–4 weeks) group (**b**). Panels (**c**, **d**) show high magnification images of the dotted boxes in the panels (**a**, **b**), respectively (Scale bar: 300 μm (**a**, **b**), 100 μm (**c**, **d**)). The arrowhead indicates the posterior capsule. (**e**) Posterior capsular thickness. Picrosirius red-stained sections of partial posterior joint capsules for each group (**f**, **g**) (Scale bar: 100 μm). (**h**) Percentage of collagen fiber in the posterior capsule measured as the ratio of the areas stained red by picrosirius red to the total areas of the posterior capsule. Data are expressed as the mean ± standard errors. *p < 0.05, **p < 0.01; n = 8. *F* femur, *T* tibia, *MM* medial meniscus, *Non-Im* mice with non-immobilized knees, *Im+/Met−* mice with immobilized knees but no metformin, *Im+/Met 0–4 weeks* mice with immobilized knees and metformin administered from 0 to 4 weeks, *Im+/Met 0–2 weeks* mice with immobilized knees and metformin administered from 0 to 2 weeks, *Im+/Met 2–4 weeks* mice with immobilized knees and metformin administered from 2 to 4 weeks.

Upon quantitative evaluation using HE staining, the posterior capsular thickness in the Im+ groups was found to be significantly greater than in the Non-Im group (p < 0.01 in all cases) (Fig. 5e). The posterior capsular thickness was found to be significantly smaller in the (Im+/Met 0–4 weeks) and (Im+/Met 0–2 weeks) group than in the (Im+/Met−) group (p < 0.01 and p < 0.01, respectively). The posterior capsular thickness in the (Im+/Met 2–4 weeks) group was not different from that in the (Im+/Met−) group. Upon quantitative evaluation using picrosirius red staining, the percentage of collagen fibers in the Im+ groups was found to be significantly greater than in the Non-Im group (p < 0.01 in all cases) (Fig. 5h). The percentage of collagen fibers in the posterior joint capsule was significantly lower in the (Im+/Met 0–4 weeks) and (Im+/Met 0–2 weeks) group than in the (Im+/Met−) group (p < 0.01 and p < 0.05, respectively). The percentage of collagen fibers in the posterior joint capsule in the (Im+/Met 2–4 weeks) group was not different from that in the (Im+/Met−) group.

#### Gene expression in the joint capsule tissue

**Figure 6:**
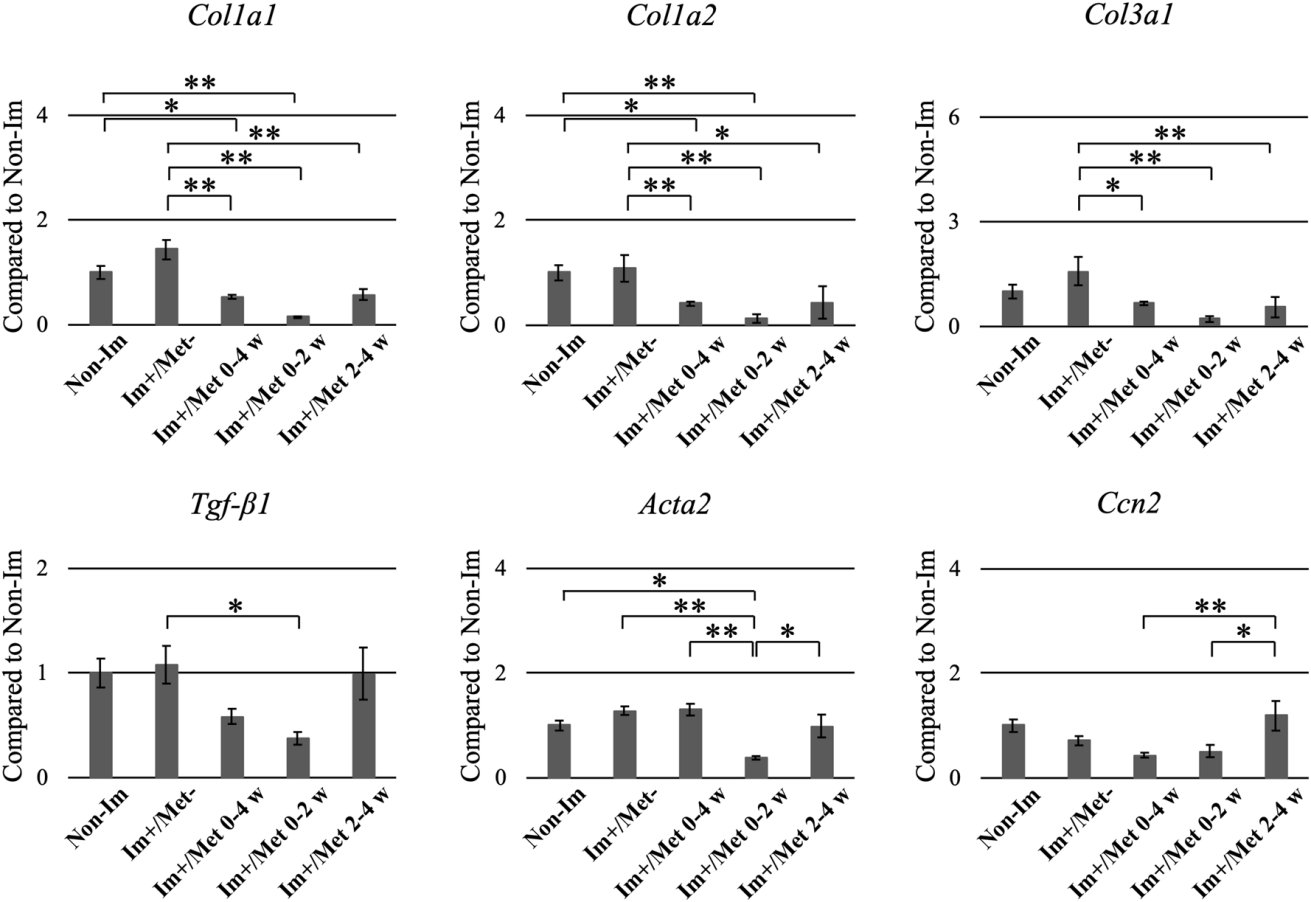
Expression analysis of the fibrosis-related genes in the knee joint capsule for the effective time of metformin administration for joint capsular fibrosis. The mRNA expression levels of *Col1a1*, *Col1a2*, *Col3a1*, *Tgf-β1*, *Acta2*, and *Ccn2* were normalized to that of *GAPDH*. Data are expressed as the mean ± standard errors. *p < 0.05, **p < 0.01; n = 8. *Col1a1*, collagen type IA1; *Col1a2*, collagen type IA2; *Col3a1*, collagen type IIIA1; *Tgf-β1*, transforming growth factor beta 1; *Acta2*, actin alpha 2; *Ccn2*, cellular communication network factor 2; *Gapdh*, glyceraldehyde-3-phosphate dehydrogenase; Non-Im, mice with non-immobilized knees; Im+/Met−, mice with immobilized knees but no metformin; Im+/Met 0–4 weeks, mice with immobilized knees and metformin administered from 0 to 4 weeks; Im+/Met 0–2 weeks, mice with immobilized knees and metformin administered from 0 to 2 weeks; Im+/Met 2–4 weeks, mice with immobilized knees and metformin administered from 2 to 4 weeks.

The expression of *Col1a1*, *Col1a2*, and *Col3a1* in the three metformin-administered groups was significantly lower than that in the (Im+/Met−) group (Fig. 6). Furthermore, the expression of *Tgf-β1* and *Acta2* in the (Im+/Met 0–2 weeks) group was significantly lower than that in the (Im+/Met−) group. In the (Im+/Met 0–2 weeks) group, the expression of *Col1a1*, *Col1a2*, and *Acta2* was significantly lower than that in the Non-Im group, that of *Acta2* was significantly lower than that in the (Im+/Met 0–4 weeks) group, and that of *Acta2* and *Ccn2* was significantly lower than that in the (Im+/Met 2–4 weeks) group. The expression of *Ccn2* in the (Im+/Met 2–4 weeks) group was significantly higher than that in the (Im+/Met 0–4 weeks) group.

#### Immunohistochemical analysis

**Figure 7:**
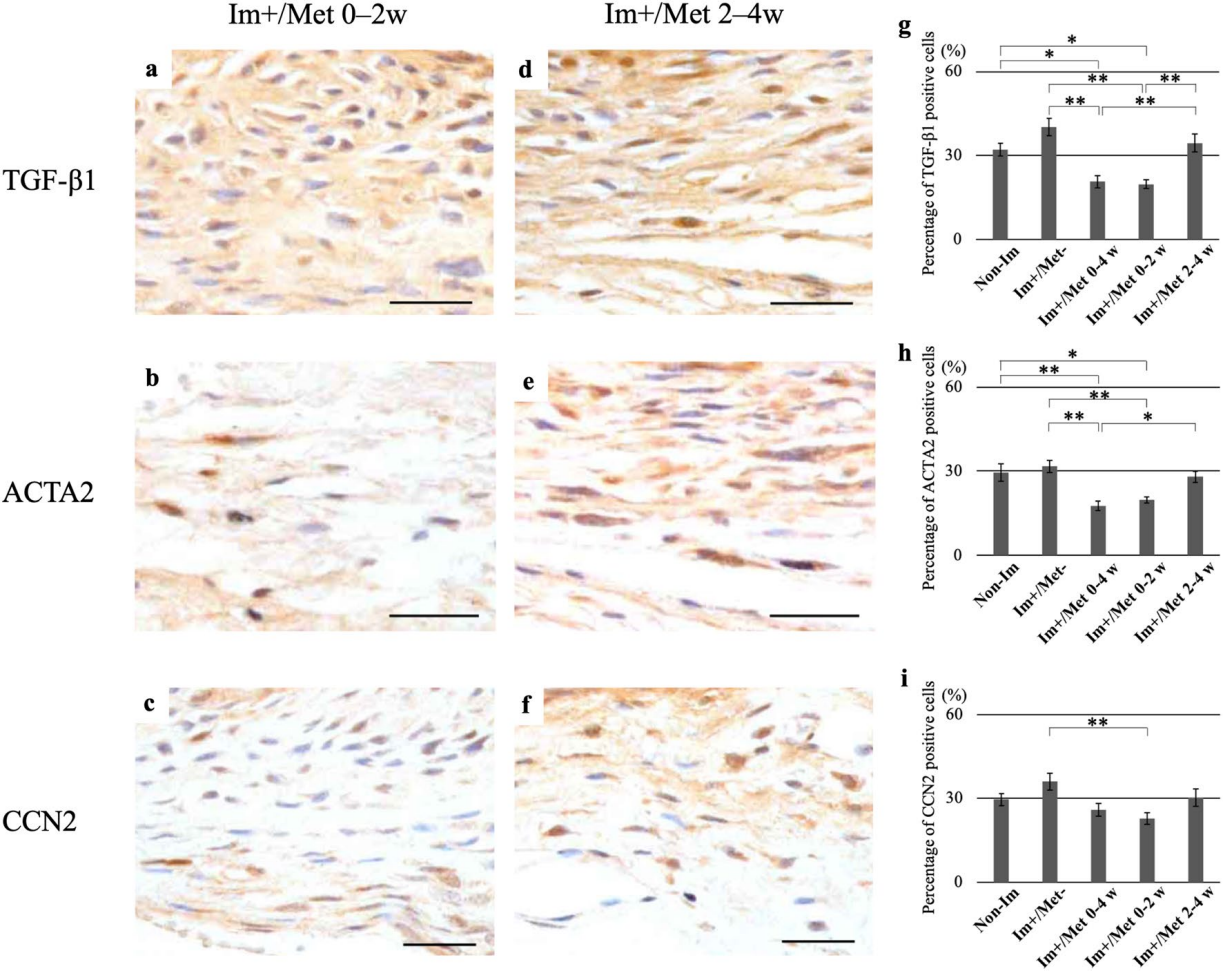
Immunohistochemical analysis for the effective time of metformin administration for joint capsular fibrosis. Immunohistochemical staining for TGF-β1, ACTA2, and CCN2 in the posterior capsule. Microscopic images of specimens immunostained for TGF-β1, ACTA2, and CCN2 with DAB and counterstained with hematoxylin in the (Im+/Met 0–2 weeks) group (**a**–**c**); and (Im+/Met 2–4 weeks) group (**d**–**f**) (Scale bar: 50 μm). Percentage of TGF-β1-positive cells (**g**), ACTA2-positive cells (**h**), and CCN2-positive cells (**i**) to the total number of cells in the posterior joint capsule. Data are expressed as the mean ± standard errors. *p < 0.05, **p < 0.01; n = 5. *TGF-β1* transforming growth factor beta 1, *ACTA2* actin alpha 2, *CCN2* cellular communication network factor 2, *Non-Im* mice with non-immobilized knees, *Im+/Met−* mice with immobilized knees but no metformin, *Im+/Met 0–4 weeks* mice with immobilized knees and metformin administered from 0 to 4 weeks, *Im+/Met 0–2 weeks* mice with immobilized knees and metformin administered from 0 to 2 weeks, *Im+/Met 2–4 weeks* mice with immobilized knees and metformin administered from 2 to 4 weeks, *DAB* 3,3′-diaminobenzidine.

The percentage of TGF-β1- and ACTA2-positive cells in the posterior capsule was significantly lower in the (Im+/Met 0–4 weeks) and (Im+/Met 0–2 weeks) group than in the (Im+/Met−) group (p < 0.01 in all cases) (Fig. 7g,h). Furthermore, the percentage of TGF-β1- and ACTA2-positive cells in the (Im+/Met 0–4 weeks) and (Im+/Met 0–2 weeks) groups was significantly lower than the Non-Im group (p < 0.05, p < 0.05, p < 0.01, and p < 0.05, respectively). The percentage of CCN2-positive cells in the posterior capsule was significantly lower in only the (Im+/Met 0–2 weeks) group than in the (Im+/Met−) group (p < 0.01) (Fig. 7i). The percentage of TGF-β1-, ACTA2-, and CCN2-positive cells in the posterior joint capsule in the (Im+/Met 2–4 weeks) group was not different from that in the (Im+/Met−) group (Fig. 7g–i).

## Discussion

In this study, we histologically and genetically described the effect of metformin treatment on joint capsular fibrosis induced by mouse knee joint immobilization. Metformin is one of the most extensively used oral anti-diabetic drugs for patients with type 2 diabetes. Recent studies have revealed an additional biological effect of metformin, a suppressive effect on tissue fibrosis in organs such as the heart, lungs, kidneys, and skin^14–21^. Herein, we investigated whether metformin plays a role in suppressing the fibrosis of joint capsule in joint contracture, where fibrosis of the joint capsule is one of the major etiologies^13^. We demonstrated that metformin treatment can suppress fibrosis of the joint capsule induced by mouse knee joint immobilization, thus indicating the potential of metformin as a novel therapeutic drug for joint contractures.

In the histological evaluation of this study, we evaluated the thickness of the posterior joint capsules and the density of collagen fibers in the posterior joint capsules. We observed that these parameters in the immobilized knee joint were greater than those in the non-immobilized knee joint, in agreement with previous studies^22,23^. Additionally, they were smaller in the metformin-administered group than in the non-administered group. Therefore, we demonstrated that metformin treatment reduces the thickness of the posterior joint capsules and the density of the collagen fibers in them, which is caused by immobilization of the joint.

The AMPK pathway is the most well-known metformin-mediated pathway^20^. AMPK is a serine/threonine kinase and an intracellular metabolic sensor. Pharmacological action of metformin is mediated via the phosphorylation of AMPK^24^, which not only maintains cellular energy homeostasis via lipid and glucose metabolism but also regulates a wide array of cell functions^25,26^. Some recent studies demonstrated that tissue fibrosis in various organs were efficiently suppressed by metformin-mediated AMPK activation^15,16,18,20,21^.

In previous studies, it has been reported that metformin, which has an AMPK-activating effect, suppresses tissue fibrosis by inhibiting TGF-β signaling in various fibrotic diseases^14–16,18,20,21^. TGF-β1 promotes fibroblast to myofibroblast differentiation, myofibroblast proliferation, and production of collagen, and is therefore considered to be the most important cytokine in fibrotic processes^27^. The induction and activation of TGF-β1 are most often observed in experimental models of tissue fibrosis, where remarkable fibrotic changes are induced in tissues overexpressing TGF-β1. Acta2 is a marker of myofibroblasts, and activation of myofibroblasts promotes tissue fibrosis^28^. Ccn2 has been recognized as a marker of fibrosis because it is consistently associated with fibrotic remodeling in various organ systems^29^. Furthermore, Ccn2 is involved in the onset and progression of tissue fibrosis in various organs^30^. It has also been reported that Ccn2 may be a potential downstream mediator for TGF-β signaling in fibroblasts and part of the pro-fibrotic effects of TGF-β are mediated through the upregulation of Ccn2^27,31^. The activation of AMPK by metformin significantly reduced TGF-β1-induced collagen production and *Acta2* expression^21,32^. Therefore, considering the involvement of TGF-β signaling, we decided to evaluate the expression levels of TGF-β and its downstream fibrosis-related genes in this study.

Expression of fibrosis-related genes measured herein is previously shown to be increased in experimental animal models in which the knee joint is immobilized^13,22,33,34^. In contrast, it has been reported that their expression is suppressed in fibrotic tissues treated with metformin^19–21,32^. In this study, the mRNA expression of some fibrosis-related genes 2 and 4 weeks after immobilization were significantly lower in the metformin-treated group than in the non-treated group, among the immobilized groups. The protein levels of TGF-β1, ACTA2, and CCN2 were also significantly lower in the metformin-treated group at both 2 and 4 weeks, as shown in the immunohistochemical analysis. Although TGF-β1 itself was not significantly upregulated by joint immobilization compared to non-immobilization at both mRNA and protein levels, the expression of TGF-β1 at protein and mRNA levels showed similar variation patterns to other fibrosis-related genes. Specifically, in comparison between the (Im+/Met−) group and the metformin-treated group, TGF-β1 expression at mRNA (Figs. 3, 6) and protein (Figs. 4, 7) levels was significantly reduced in metformin treatment, as was the case with other fibrosis-related genes. Previous studies reporting the suppressing effect of metformin on tissue fibrosis also significantly reduced the expression of the fibrosis-related genes, including TGF-β in fibrotic tissue treated with metformin^19–21,32^. Therefore, the inhibition of TGF-β signaling may be the main mechanism by which metformin suppresses fibrosis of joint capsules, although a detailed analysis of the mechanism by which metformin suppresses the fibrosis of joint capsules was not performed in this study.

Furthermore, this study suggested that the most effective time for administration of metformin on joint capsular fibrosis may be early after joint immobilization; a delayed administration of 2 weeks or more after joint immobilization may be less effective. From the results of qRT-PCR, the expression of fibrosis-related genes in two Im groups tended to peak at 2 weeks and decrease at 4 weeks. Similar gene expression patterns have been reported in other animal models of joint contracture^34^. Owing to the expression pattern of the fibrosis-related genes in the joint contracture model, it was considered that it may be important to administer metformin as early as 2 weeks after joint immobilization. Thus, to investigate the importance of the timing of metformin administration, two groups, with the first group receiving metformin for 0–2 weeks and the second group receiving metformin for 2–4 weeks after joint immobilization, were added.

From the histological evaluations using HE staining and picrosirius red staining, we observed that the first group showed the same effect of suppressing the fibrosis of joint capsule as the group treated with metformin for the entire 4 weeks, but the second group did not show this effect. Similar results were obtained in the immunohistochemical evaluations. In the evaluation of gene expression in the joint capsule, the expression of fibrosis-related genes was most suppressed in the first group compared with that in the non-treated group. Therefore, we suggest that administration of metformin from an early period following joint immobilization is more effective for suppressing joint capsular fibrosis, and administration of metformin after a certain delayed time cannot be expected to have this effect. In other words, we suggest that it is important to administer metformin early after the events that induce joint capsular fibrosis, that is, when joint capsular fibrosis begins to form (Im+/Met 0–2 weeks), and it is less effective to administer metformin after 2 weeks of joint immobilization with already-formed joint capsular fibrosis (Im+/Met 2–4 weeks). In most studies reporting the suppressing effect of metformin on tissue fibrosis, metformin was administered early after treatment for inducing tissue fibrosis or early after the onset of tissue fibrosis^14–16,19,20^. Rangarajan et al. performed a study investigating the effect of metformin on lung fibrosis at two different times, 10–18 days and 3–5 weeks after bleomycin administration, in a mouse model of bleomycin-induced lung fibrosis^18^. The result of this study showed that the suppressive effect of metformin on lung fibrosis was observed not only at the early times but also at delayed times; however, the results between the more effective time points were not compared. Our study is the first to investigate the difference in the suppressive effect of metformin on tissue fibrosis depending on the time of administration. Via this experiment using a mouse knee immobilization model, it was found that the effective time of metformin administration in the treatment of joint capsular fibrosis was early after joint immobilization; a delayed administration of 2 weeks or more after joint immobilization was less effective. Although no studies have been reported on the effect of metformin on joint contracture in humans, there may be an effective time of administration of metformin in humans as well as in mice. Regarding this point, we surmise that further clinical research is warranted in the future.

There are two limitations to this study. First, we evaluated joint contracture in only the joint capsule. It has been reported there are arthrogenic and myogenic components that cause immobilization-induced joint contractures^10^. However, it was not possible to evaluate all factors associated with joint contracture at the same time in each experiment. Therefore, we focused on the joint capsule, which is the most important of the joint components associated with the irreversibility of joint contracture^11,12,35^, as the assessment site for all evaluation items in this study. However, it is necessary to also evaluate the effect of metformin on each factor associated with joint contracture other than joint capsule in the future. Second, the evaluation was performed at 2 and 4 weeks after the start of the experiment. As the joint contracture model used in this study envisions joint contracture as a complication of the treatment of musculoskeletal diseases due to external fixation of joints, we decided to set the endpoint for this study from 2 to 4 weeks, which is a frequently set period for external fixation for the purpose of local rest. However, it is also necessary to evaluate the effects of metformin on joint contracture due to long-term immobilization in the future.

In conclusion, this study demonstrated that metformin treatment can suppress fibrosis of the joint capsule in a knee joint contracture mouse model, thus indicating the potential of metformin as a novel therapeutic drug for joint capsular fibrosis in joint contractures. Furthermore, it was found that the effective time of metformin administration in the treatment of joint capsular fibrosis was early after the events that induce fibrosis, and that a delayed administration of 2 weeks or more after joint immobilization was less effective.

## Materials and methods

### Effect of metformin on prevention of joint contracture in a mouse model with knee immobilization

#### Animals

In this study, 10-week-old C57BL/6 J male mice (22–27 g; purchased from Charles River Laboratories, Tokyo, Japan and Clea, Tokyo, Japan) were used. The mice obtained from two different locations were distributed evenly to each group. As it has been reported that estrogen suppresses tissue fibrosis^36^, we used only male mice, which are less affected by estrogen, in this study. Mice were housed in standard cages in a temperature-controlled room (20–25 °C) with a 12 h light/dark cycle. The mice were provided standard rodent food and water ad libitum. The study was approved by the animal experimentation committee of University of Occupational and Environmental Health (AE18-013), and all experiments were performed in accordance with the guidelines of the University of Occupational and Environmental Health ethics committee. This study was carried out in compliance with the ARRIVE guidelines.

#### Study design

The mice were divided into three groups: a group in which the knees were not immobilized and metformin not administered (Non-Im), a group in which the knees were immobilized and metformin not administered (Im+/Met−), and a group in which the knees were immobilized and metformin administered (Im+/Met+). In the two Im+ groups, the immobilization of the knee of right hind legs was performed by external fixation using splint and tape under general anesthesia, as described previously^22^. The angle of knee joint immobilization was standardized by setting the angle of the splint used for joint immobilization to 120°. A cocktail of midazolam (0.2 mg/kg), butorphanol tartrate (1.0 mg/kg), and medetomidine hydrochloride (0.3 mg/kg) was intraperitoneally injected to anesthetize the animals during surgery. In the Non-Im group, the right hind legs were used for analysis as in the Im+ groups. In Met+ group, metformin (65 mg/kg) dissolved in saline was administered intraperitoneally every other day during the experimental period after immobilization of the knee. The metformin dosage was referred from previous studies^18^. The test groups (n = 96) consisted of the Non-Im group, (Im+/Met−) group, and (Im+/Met+) group, with 32 mice in each group. The experimental period was 2 weeks (n = 16) and 4 weeks (n = 16). In each experimental period, half the mice were assigned for the evaluation of gene expression (n = 8) and the other half for the evaluation of histology (n = 8) and immunohistochemistry (n = 5), in each group (Fig. 1a). Other than the immobilized part, the animals were left free to move inside the cage during the experimental period. Following the completion of each experimental period, mice were euthanized by cervical dislocation under anesthesia by inhalation of sevoflurane, and mice tissues required for evaluations were collected.

### Determination of effective time of metformin administration for preventing joint capsular fibrosis

To examine the difference in the effect due to the time of metformin administration, two groups with immobilized right knees and metformin administered at different times were added in this study. In the first group, metformin was administered only for the first half, i.e., 0–2 weeks of the 4-week experimental period (Im+/Met 0–2 weeks), and in the other group, metformin was administered only for the second half, i.e., 2–4 weeks of the 4-week experimental period (Im+/Met 2–4 weeks). The experimental period was 4 weeks in both groups, and the number of mice and the analysis method were the same as in the three groups in the previous section. Data from the (Im+/Met+) group from the first experiment was reused for the (Im+/Met 0–4 weeks) group for this experiment. Differences among the five groups: Non-Im group at 4 weeks, (Im+/Met−) group at 4 weeks, (Im+/Met 0–4 weeks), (Im+/Met 0–2 weeks), and (Im+/Met 2–4 weeks), were analyzed in this experiment (Fig. 1b).

### Gene expression analysis of the joint capsule

In this experiment, the expression levels of the fibrosis-related genes such as *Col1a1*, *Col1a2*, *Col3a1*, *Tgf-β1*, *Acta2* and *Ccn2* were evaluated in the joint capsule tissues collected from the knees of each group. The right hind limb was dissected at the hip level after euthanizing the animals, and the skin and muscles were removed from the disconnected right hind limb to expose the joint capsule. Then, the joint capsule tissues were delicately collected.

The joint capsule tissues were placed in a 1.5 mL tube and dissolved in 500 μL of TRIzol reagent (Invitrogen, Carlsbad, CA, USA). After mechanical homogenization, the mixture was centrifuged at 2000×*g* for 4 min at 4 °C. The supernatant was transferred to a new tube and chloroform was added into it. After being stirred, the mixture was centrifuged at 12,000×*g* for 15 min at 4 °C. The supernatant was transferred to a new tube, and the total RNA was extracted with an acid guanidinium thiocyanate–phenol–chloroform method. RNA integrity and purity were assessed by calculating the ratio of absorption at 260/280. The total RNA was reverse-transcribed in a 20 μL reaction volume using the iScript cDNA Synthesis Kit (Bio-Rad Laboratories, Hercules, CA, USA), according to the manufacturer’s recommendations.

Quantitative real-time polymerase chain reaction (qRT-PCR) analysis was performed by the delta–delta CT method using a CFX Connect Real-Time System (Bio-Rad) with CFX Manager Software version 3.1 (Bio-Rad). The qRT-PCR analyses for the genes, such as *Col1a1*, *Col1a2*, *Col3a1*, *Tgf-β1*, *Acta2*, *Ccn2*, and glyceraldehyde-3-phosphate dehydrogenase (*Gapdh*), were performed in a final reaction volume of 10 μL with 1.0 ng of cDNA, 2.0 μM primers, and 5 μL of iQ SYBR Green Supermix (Bio-Rad). The primers used in this study were designed using the Primer3 software (http://frodo.wi.mit.edu/primer3/). The sequences of the oligonucleotides used in this study are shown in Table 1. The expression level of each target gene was normalized to the expression levels of the *Gapdh* transcripts^37^. Gene expression analysis was performed using eight mice in each group.

**Table 1:** Polymerase chain reaction primer sequences.

Gene name	Gene bank	Nucleic acid sequences	
*Gapdh*	NM_001289726.1	Upstream	AACTTTGGCATTGTGGAAGG
		Downstream	GGATGCAGGGATGATGTTCT
*Col1a1*	NM_007742.4	Upstream	GAGCGGAGAGTACTGGATCG
		Downstream	GTTCGGGCTGATGTACCAGT
*Col1a2*	NM_007743.3	Upstream	GTGTTCAAGGTGGCAAAGGT
		Downstream	GACCGAATTCACCAGGAAGA
*Col3a1*	NM_009930.2	Upstream	ACCAAAAGGTGATGCTGGAC
		Downstream	GACCTCGTGCTCCAGTTAGC
*Tgf-β1*	NM_011577.2	Upstream	GACTCTCCACCTGCAAGACC
		Downstream	GACTGGCGAGCCTTAGTTTG
*Acta2*	NM_007392.3	Upstream	TGTGCTGGACTCTGGAGATG
		Downstream	GAAGGAATAGCCACGCTCAG
*Ccn2*	NM_010217.2	Upstream	CACTCTGCCAGTGGAGTTCA
		Downstream	GTAATGGCAGGCACAGGTCT

*Gapdh*, glyceraldehyde-3-phosphate dehydrogenase; *Col1a1*, collagen type IA1; *Col1a2*, collagen type IA2; *Col3a1*, collagen type IIIA1; *Tgf-β1*, transforming growth factor beta 1; *Acta2*, actin alpha 2; *Ccn2*, cellular communication network factor 2.

### Histology and immunohistochemistry

Following euthanasia, the right hind legs were fixed with 4% paraformaldehyde (pH 7.4) for about 36 h at 4 °C. The samples were decalcified in K-CX (FALMA, Tokyo, Japan) for 3 h at 20–25 °C, and neutralized with 5% sodium sulfate solution (Wako Pure Chemical Corporation, Osaka, Japan). The samples were embedded in paraffin and the knee angles were set to 60° in all groups. Then, the samples were cut into 5-μm sagittal sections using a microtome. Sections were cut from the medial edge of the knee, and serial sections were collected after the medial meniscus was separated anteriorly and posteriorly. Three sections were selected for every five sections from the first of the serial sections and stained with HE. We measured the posterior capsular thickness using the All-in-One Fluorescence Microscope BZ-X710 (Keyence, Osaka, Japan) and BZ-X Analyzer (Keyence). As previously described^22^, the posterior capsular thickness was measured behind the meniscus, which was the portion from the extension of the perpendicular line drawn from the apex of the meniscus to its trailing edge. It was measured as the distance from the point in contact with the meniscus as the anterior edge to the last surface of the continuous collagen tissue as the posterior edge. This analysis was performed using three slides per mouse, with eight mice in each group.

Furthermore, three additional sections were stained with picrosirius red (ab150681, Abcam, Cambridge, UK), according to the manufacturer’s recommendations, to evaluate the density of the collagen fibers as the ratio of the area stained red by picrosirius red to the total area of the posterior capsule, as previously described^22^. Each area was quantified using the ImageJ 1.46r software package (http://imagej.nih.gov/ij; US National Institute of Health, Bethesda, ML). This analysis was performed in eight mice in each group, using three slides per mouse.

For immunohistochemistry, sections were deparaffinized and washed in phosphate buffered saline (PBS). Endogenous peroxidase was inactivated with 3% H_2_O_2_/methanol for 10 min, and nonspecific binding of immunoglobulins was blocked by incubation for 20 min with Protein Block Serum-Free (DAKO, Carpinteria, CA, USA). The slides were washed again in PBS, incubated with a rabbit anti-mouse TGF-β1 polyclonal antibody (ab92486, Abcam), a rabbit anti-mouse ACTA2 polyclonal antibody (ab5694, Abcam) or a rabbit anti-mouse CCN2 polyclonal antibody (ab6992, Abcam) diluted with PBS at the recommended concentration for 2 h at 20–25 °C, and then rinsed in PBS. Rabbit immunoglobulin G (IgG) isotype control (bs-0295P, Funakoshi Co., Ltd, Tokyo, Japan) was used as a negative control to confirm that the reaction of the primary antibody to the target was specific. The slides were incubated with HISTOFINE simple stain mouse MAX-PO(R) (Nichireivioscience Inc., Tokyo, Japan) for 30 min at 20–25 °C. After rinsing with PBS, the final detection was performed using DAB IHC Substrate (GenWay Biotech, San Diego, CA) for 30 s. Slides were counterstained with hematoxylin.

Two posterior capsule sites from each slide were photographed at 400× magnification using the All-in-One Fluorescence Microscope BZ-X710 (Keyence). In each image, the number of total, TGF-β1-positive, ACTA2-positive, or CCN2-positive cells in the posterior joint capsule, excluding the vascular endothelial, adipocyte, and meniscal cells, was counted using the ImageJ 1.46r software package. For counting the total cell number, the threshold was set to 120 px for each image. When the number of TGF-β1-positive, ACTA2-positive, or CCN2-positive cells were counted, the threshold was set to 70 px. Then, the ratio of TGF-β1-positive, ACTA2-positive, or CCN2-positive cells to the total number of cells was calculated. The first five consecutive mice were automatically selected from the eight mice used for histological analysis in each group and immunohistochemical analysis was performed in two different areas from one knee joint paraffin section of each selected mouse.

### Statistical analysis

All results are expressed as the means ± standard errors. One-way analysis of variance (ANOVA) with Tukey post hoc analysis was used to detect the differences in the conditions between three groups: Non-Im group, (Im+/Met−) group, and (Im+/Met+) group, and five groups: Non-Im group, (Im+/Met−) group, (Im+/Met 0–4 weeks) group, (Im+/Met 0–2 weeks) group, and (Im+/Met 2–4 weeks) group. Results were considered statistically significant at p-values of p < 0.05. Calculations were performed with IBM SPSS Statistics version 22 software (IBM Corp., Armonk, NY, USA) on a Macintosh computer (Apple Corp., Cupertino, CA, USA).

## Data Availability

All data generated or analyzed during this study are included in this published article.
